# Examining the Impact of Ertugliflozin on Cardiovascular Outcomes in Patients with Diabetes and Metabolic Syndrome: A Systematic Review of Clinical Trials

**DOI:** 10.3390/ph17070929

**Published:** 2024-07-11

**Authors:** Silvius Alexandru Pescariu, Ahmed Elagez, Balaji Nallapati, Felix Bratosin, Adina Bucur, Alina Negru, Laura Gaita, Ioana Mihaela Citu, Zoran Laurentiu Popa, Paula Irina Barata

**Affiliations:** 1 Department of Cardiology, Victor Babes University of Medicine and Pharmacy, 300041 Timisoara, Romania; pescariu.alexandru@umft.ro (S.A.P.); eivanica@yahoo.com (A.N.); 2 Department of General Medicine, Misr University for Science & Technology, Giza 3236101, Egypt; ahmeddmahmouudd@gmail.com; 3 Department of General Medicine, Katuri Medical College and Hospital, Katuri City 522019, India; dr.balajinallapati@gmail.com; 4 Department of Infectious Disease, Victor Babes University of Medicine and Pharmacy, 300041 Timisoara, Romania; felix.bratosin@umft.ro; 5 Department III Functional Sciences, Division of Public Health and Management, University of Medicine and Pharmacy Victor Babes Timisoara, 300041 Timisoara, Romania; 6 Second Department of Internal Medicine, University of Medicine and Pharmacy Victor Babes Timisoara, 300041 Timisoara, Romania; gaita.laura@umft.ro; 7 First Department of Internal Medicine, University of Medicine and Pharmacy Victor Babes Timisoara, 300041 Timisoara, Romania; citu.ioana@umft.ro; 8 Department of Obstetrics and Gynecology, University of Medicine and Pharmacy Victor Babes Timisoara, 300041 Timisoara, Romania; popa.zoran@umft.ro; 9 Center for Research and Innovation in Precision Medicine of Respiratory Diseases, University of Medicine and Pharmacy Victor Babes Timisoara, 300041 Timisoara, Romania; barata.paula@student.uvvg.ro; 10 Department of Physiology, Faculty of Medicine, “Vasile Goldis” Western University of Arad, 310025 Arad, Romania

**Keywords:** cardiology, diabetes, systematic review, heart failure

## Abstract

Cardiovascular diseases (CVDs) constitute a significant cause of morbidity and mortality globally, particularly among individuals with type 2 diabetes mellitus (T2DM). Ertugliflozin, a Sodium-Glucose Co-transporter-2 (SGLT2) inhibitor, is hypothesized to confer cardiovascular protection; however, long-term follow-up studies are necessary to support the hypothesis. This systematic review was conducted to evaluate the cardiovascular effects of ertugliflozin in diabetic versus non-diabetic cohorts, focusing on major adverse cardiovascular events (MACEs), hospitalizations for heart failure, and cardiovascular mortality. Adhering to PRISMA guidelines, the review encompassed studies indexed in PubMed, Scopus, and Web of Science up to March 2024. Eligibility was restricted to studies involving T2DM patients undergoing ertugliflozin treatment with reported outcomes relevant to cardiovascular health. Out of 767 initially identified articles, 6 met the inclusion criteria. Data concerning hazard ratios (HR) and confidence intervals (CI) were extracted to compare the effects of ertugliflozin with those of a placebo or other standard therapies. The collective sample size across these studies was 8246 participants. Ertugliflozin was associated with a significant reduction in hospitalizations for heart failure relative to a placebo (HR 0.70, 95% CI 0.54–0.90, *p* < 0.05). Furthermore, when combined with metformin, ertugliflozin potentially reduced MACEs (HR 0.92, 95% CI 0.79–1.07), although this finding did not reach statistical significance. Importantly, for patients with pre-existing heart failure, ertugliflozin significantly decreased the exacerbations of heart failure (HR 0.53, 95% CI 0.33–0.84, *p* < 0.01). Overall, ertugliflozin markedly reduces hospitalizations due to heart failure in T2DM patients and may improve additional cardiovascular outcomes. These results endorse the integration of ertugliflozin into therapeutic protocols for T2DM patients at elevated cardiovascular risk and substantiate its efficacy among SGLT2 inhibitors. Continued investigations are recommended to delineate its long-term cardiovascular benefits in diverse patient populations, including the potential impact on arrhythmias.

## 1. Introduction

Cardiovascular diseases (CVDs) remain the leading cause of mortality worldwide, accounting for approximately 17.9 million deaths annually, a figure that represents a significant public health challenge [1]. The burden of CVD is particularly pronounced among individuals with metabolic disorders such as type 2 diabetes mellitus (T2DM), where the risk of developing cardiovascular complications is markedly elevated [2,3]. Diabetes itself is a global epidemic, with current estimates indicating that over 460 million adults live with the condition, a number expected to rise past 700 million by 2045 [4,5].

The pathophysiological link between T2DM and CVD is complex, involving a multifaceted interplay of hyperglycemia, insulin resistance, and a host of metabolic derangements, including dyslipidemia and hypertension [6,7]. These factors contribute to a heightened state of inflammation and increased atherosclerotic burden, propelling the progression of cardiovascular pathology [8]. Accordingly, the management of hyperglycemia in diabetic patients is aimed not only at controlling blood glucose levels but also at mitigating cardiovascular risk, which has propelled the development of antidiabetic therapies with cardioprotective properties [9,10].

In this regard, Sodium-Glucose Cotransporter 2 (SGLT2) inhibitors have emerged as a significant advancement in the treatment landscape of T2DM, with implications for CVD management [11,12]. SGLT2 inhibitors, by facilitating glucose excretion through kidney filtration, not only improve glycemic control but also confer benefits on body weight, blood pressure, and metabolic control [13]. Emerging evidence from clinical trials suggests that SGLT2s can significantly reduce hospitalization for heart failure and may lower the incidence of major adverse cardiovascular events in patients with T2DM [14,15]. Since their initial introduction in clinical practice, the hypotheses regarding the mechanisms of action of SGLT2 inhibitors have evolved significantly: initially viewed as straightforward glycosuric agents that lower glucose levels, enhance erythropoiesis, and stimulate ketogenesis, this class of drugs is now recognized as one containing intracellular sodium-lowering compounds. This property underlies their observed cardioprotective effects, which contribute to a substantial reduction in cardiovascular events, particularly in high-risk populations. From a molecular standpoint, the administration of gliflozins to patients induces conditions akin to nutrient and oxygen deprivation, thereby triggering autophagy to uphold cellular homeostasis via diverse degradative mechanisms [16]. In recent years, SGLT2 inhibitor uses have also expanded into the realm of renal protection [17].

Ertugliflozin was approved by the U.S. Food and Drug Administration (FDA) in December 2017 and subsequently received marketing authorization from the European Medicines Agency (EMA) in March 2018 [18]. Animal studies of ertugliflozin prior to these approvals demonstrated promising outcomes, particularly in the context of glucose control and potential cardiovascular benefits [19]. In rodent models, ertugliflozin effectively reduced blood glucose levels, body weight, and visceral adiposity [20]. Moreover, these studies suggested improvements in cardiac function and structure, indicating potential protective effects against heart failure—a common complication in diabetic populations.

While other SGLT2 inhibitors have enjoyed the spotlight regarding their efficacy against heart failure, the hypothesis of this study is that ertugliflozin also provides a significant cardiovascular benefit, an effect which it would share with the other drugs in the SGLT2 inhibitor class. The primary objective of this systematic review is to evaluate the extent of cardiovascular outcomes associated with the use of ertugliflozin in the diabetic and non-diabetic population. This investigation aims to inform clinical practice and guide future research in the management of cardiovascular risk. While ertugliflozin has shown promise in initial studies for its cardiovascular benefits, comprehensive analysis is required to consolidate these findings and assess their robustness across diverse patient populations. This study can provide a thorough evaluation of existing evidence, addressing gaps in knowledge about the long-term efficacy and safety of ertugliflozin’s action on heart failure

## 2. Materials and Methods

### 2.1. Eligibility Criteria

This systematic review selected studies for inclusion based on the following criteria and the existing literature [16,17,18]: (1) patients diagnosed with type 2 diabetes mellitus (T2DM) and treated with the SGLT2 inhibitor ertugliflozin either as monotherapy or in combination with other antidiabetic agents; (2) research specifically investigating the cardiovascular outcomes associated with the use of ertugliflozin, with a particular focus on major adverse cardiovascular events, hospitalization for heart failure, and overall cardiovascular mortality; (3) studies that were defined as clinical trials; (4) studies employing validated methods or clearly defined parameters to evaluate cardiovascular outcomes, efficacy, safety profiles, and patient adherence; and (5) only peer-reviewed articles published in English.

The exclusion criteria were the following: (1) studies not involving human participants, such as in vitro or animal studies, except where preclinical data were used to support clinical findings; (2) research not specifically examining patients treated with ertugliflozin or studies that did not differentiate the effects of ertugliflozin from other SGLT2 inhibitors; (3) studies that failed to provide clear, quantifiable outcomes related to cardiovascular health or lacked sufficient detail for a comprehensive assessment; (4) grey literature, including non-peer-reviewed articles, preprints, conference abstracts, general reviews, commentaries, and editorials, to avoid potential biases and inconsistencies; (5) studies deemed low quality based on predetermined, quantifiable metrics; and (6) studies with different designs other than clinical trials.

### 2.2. Information Sources

The primary information sources for this systematic review were the electronic databases PubMed, Scopus, and the Web of Science. The literature search specifically targeted publications up to the initial search date, 17 March 2024. The search was designed to capture a wide array of studies, including those that evaluated clinical outcomes, patient demographics, treatment modalities, and the long-term cardiovascular effects associated with ertugliflozin.

The PICOS statement of the following study was considered as follows:

P (Population): Patients diagnosed with type 2 diabetes mellitus (T2DM), including both diabetic and non-diabetic individuals being treated with ertugliflozin either as monotherapy or in combination with other antidiabetic agents.

I (Intervention): Use of the Sodium-Glucose Cotransporter 2 (SGLT2) inhibitor, ertugliflozin, focusing on its impact on cardiovascular outcomes.

C (Comparison): Comparison of ertugliflozin with placebo or other standard diabetes treatments, assessing differential impacts on cardiovascular outcomes.

O (Outcomes): Primary outcomes include major adverse cardiovascular events (MACEs), such as myocardial infarction, stroke, cardiovascular death, coronary revascularization, hospitalization for unstable angina, sudden cardiac arrest, significant arrhythmias, and changes in renal function measured by eGFR levels. Secondary outcomes involve patient demographic data, duration of diabetes, baseline HbA1c levels, lipid profiles, and overall patient safety and efficacy of the treatment.

S (Study Design): Randomized controlled trials (RCTs), observational studies, cohort studies, case-control studies, and cross-sectional studies published in peer-reviewed journals and written in English.

### 2.3. Search Strategy

The search strategy for this systematic review was crafted using keywords and phrases directly relevant to the study’s objectives, focusing on the interplay between ertugliflozin, cardiovascular disease, and diabetes management. The keywords included “type 2 diabetes mellitus”, “T2DM”, “cardiovascular disease”, “CVD”, “heart failure”, “major adverse cardiovascular events”, “blood pressure”, “SGLT2 inhibitors”, “ertugliflozin”, “clinical outcomes”, “cardiovascular mortality”, “hospitalization”, “glycemic control”, “metabolic effects”, “patient safety”, and “efficacy”.

To maximize the precision and coverage of the literature search, these terms were combined using Boolean operators (AND, OR, NOT) along with relevant Medical Subject Headings (MeSH) to refine the search further. The formulated search string included combinations such as: ((“diabetes” OR “type 2 diabetes mellitus” OR “T2DM”) AND (“ertugliflozin” OR “SGLT2 inhibitors”) AND (“cardiovascular disease” OR “heart failure” OR “heart disease”) AND (“clinical outcomes” OR “hospitalization” OR “cardiovascular mortality”) AND (“safety” OR “efficacy”)).

### 2.4. Selection Process

In line with the Preferred Reporting Items for Systematic Reviews and Meta-Analyses (PRISMA) guidelines [21], the selection process of this systematic review was structured to ensure transparency and reproducibility. Initially, all the retrieved records underwent an independent screening by two reviewers (S.A.P. and F.B.) to ascertain their eligibility according to the predefined inclusion and exclusion criteria. Any discrepancies between the reviewers at this stage were addressed through a consensus meeting or, if required, by consulting a third reviewer. The protocol for this review, including detailed methodologies and criteria, has been registered and is publicly accessible on the Open Science Framework (OSF) with the registration code osf.io/ve7q3.

### 2.5. Data Collection Process

The data collection process commenced with the removal of duplicate entries from the initial dataset. Subsequently, two independent reviewers (S.A.P. and F.B.) performed a detailed screening of the abstracts to evaluate the relevance of each study against the established inclusion and exclusion criteria. In cases of discrepancies between the reviewers, discussions were held to reach a consensus, and if necessary, a third reviewer was consulted to make a final determination.

### 2.6. Data Items

The primary outcomes assessed included major adverse cardiovascular events (MACEs), such as myocardial infarction (heart attack), stroke, cardiovascular death, coronary revascularization, hospitalization for unstable angina, sudden cardiac arrest, significant arrhythmias, and hospitalization due to heart failure, as well as changes in renal function, quantified through estimated Glomerular Filtration Rate (eGFR) levels. Specific hazard ratios (HR) and confidence intervals (CI) were recorded to measure the effect size of ertugliflozin compared to various controls, including a placebo and other standard treatments. Sensitivity analyses were performed in the case of missing data, in order to estimate the impact of the missing data.

Secondary outcomes involved patient demographic data, such as age, body mass index (BMI), and race, details on the duration of diabetes, and baseline HbA1c levels; lipid profiles were also collected to further contextualize the impact of ertugliflozin on long-term glucose control and lipid metabolism. We also collected data on study design, quality, and geographic location from the referenced studies. Patient characteristics, including sample size, age distribution, and comparison groups, were documented to understand the population diversity and the comparative efficacy of the treatment under different demographic settings.

We utilized standardized data extraction forms designed to systematically capture all relevant data elements from each RCT, including study design, participant demographics, intervention details, outcomes, and any reported biases. To ensure reliability and validity in our data extraction process, these forms were pilot tested on a subset of studies before full implementation. The pilot testing helped refine the forms based on the initial findings, allowing for adjustments that enhanced the accuracy and consistency of the data collected across all the studies.

### 2.7. Risk of Bias and Quality Assessment

Initially, the quality of clinical trials was evaluated using the Cochrane tool [22]. This comprehensive tool evaluates several crucial factors including random sequence generation, allocation concealment, blinding of participants and personnel, blinding of outcome assessment, incomplete outcome data, selective reporting, and other biases. Each trial was independently reviewed by two researchers to mitigate any potential assessment bias, ensuring a rigorous and objective evaluation of the trial quality.

## 3. Results

### 3.1. Study Selection and Study Characteristics

**Figure 1:**
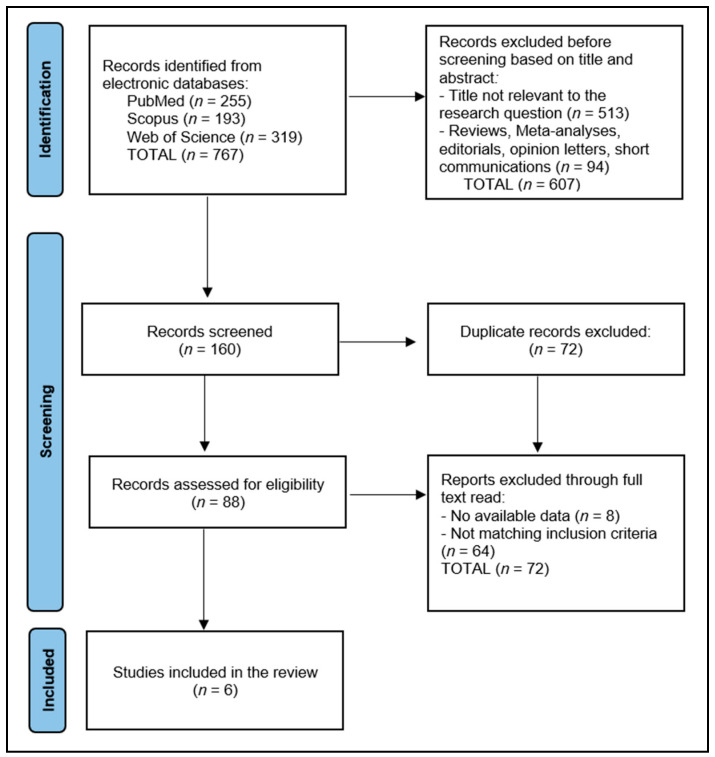
PRISMA flow diagram.

A total of 767 articles were identified according to the initial search, of which 72 duplicate entries were eliminated, 607 records were excluded before screening based on their titles and abstracts, and 72 articles were excluded after a full read for not matching the inclusion criteria or having no available data. The systematic review included a total of six studies in the final analysis, delineated in Figure 1, spanning a period from 2017 to 2023.

The analysis of the study characteristics from six research articles [23,24,25,26,27,28], as detailed in Table 1, covered a period from 2020 to 2023. These studies assessed the impact of the SGLT2 inhibitor ertugliflozin on cardiovascular outcomes in patients with type 2 diabetes. All six studies employed a randomized clinical trial design and were recognized for their high-quality methodology. The studies conducted by Cannon et al. [23] and Cosentino et al. [27] in 2020; Dagogo et al. [24] and Segar et al. [26] in 2022; and Cherney et al. [25] and Pratley et al. [28] in 2023 were all international trials, highlighting the global interest and applicability of ertugliflozin in diverse patient populations.

### 3.2. Results of Individual Studies

The existing studies performed subgroup and post hoc analyses of patients from the VERTIS-CV trial, each author focusing on the effect of ertugliflozin on different population features. Cannon et al. [21] examined a large subgroup of 5499 patients treated with ertugliflozin versus a placebo group of 2747 patients. The mean age of the participants was 64.4 years, and the average body mass index (BMI) was 32.0 ± 5.5, suggesting a predominately overweight population. A notable 87.8% of the cohort was white. This analysis focused on overall cardiovascular safety and efficacy, providing a baseline comparison for the effectiveness of ertugliflozin in reducing major adverse cardiovascular events (MACEs) compared to a placebo.

Dagogo et al. [24] analyzed the effects of combining ertugliflozin with metformin versus ertugliflozin alone in a total of 6286 participants. This comparison aimed to identify any additional benefits of metformin in the management of cardiovascular risks. The patients had a similar age profile (mean age of 64.0 years) and BMI (32.0 ± 5.4), with 87.6% of the participants being white. The study quantitatively assessed the reduction in MACEs and hospitalizations for heart failure, comparing the combined treatment versus ertugliflozin alone.

Cherney et al. [25] focused specifically on patients with heart failure, analyzing 1605 patients treated with ertugliflozin. This group was further subdivided based on heart failure presence and absence, comparing these to their respective placebo groups. This detailed examination helped elucidate the drug’s impact on heart failure outcomes, a critical aspect given the high cardiovascular risk in the diabetic population. Segar et al. [24] also investigated a large cohort of 5499 patients, mirroring the subgroup in Cannon et al. [21], but potentially focusing on different aspects, such as the progression of renal impairment or specific cardiovascular outcomes over time. Detailed statistical results from this study would help in understanding the long-term benefits or risks associated with ertugliflozin treatment.

Cosentino et al. [27] divided their analysis among patients based on the severity of heart failure and ejection fraction (EF), creating subgroups such as HF with EF ≤ 45% and HF with EF >45%, compared against similar placebo groups. This stratification provided insights into how varying degrees of cardiac function might influence the efficacy of ertugliflozin, particularly in reducing hospitalizations or improving survival rates. Pratley et al. [26] explored age-related effects by dividing their cohort into segments based on age thresholds (≥65 years and <65 years) and further stratified by elderly (≥75 years) versus younger segments (<75 years). By detailing BMI across these age groups and comparing them to corresponding placebo groups, the study highlighted how age and body composition interact with treatment outcomes, particularly in terms of renal function and cardiovascular risk reduction, as seen in Table 2.

### 3.3. Results of Synthesis

The participants had a mean duration of diabetes ranging from 9.4 to 13.8 years, reflecting a long-term management scenario typical in type 2 diabetes populations. Across the studies, the mean or median HbA1c values were consistently around 8.2%. Lipid profiles, where reported, indicated a mixed dyslipidemia common in type 2 diabetes. For instance, Cannon et al. [21] reported a detailed lipid profile with total cholesterol at 168.9 ± 46.9 mg/dL, LDL cholesterol at 89.3 ± 38.5 mg/dL, HDL cholesterol at 43.7 ± 12.0 mg/dL, and triglycerides at 181.4 ± 119.2 mg/dL. Meanwhile, Dagogo et al. [22] noted a 75.8% prevalence of dyslipidemia in their study cohort. The eGFR showed variability across the subgroups, ranging from 46.0 to 78.1 mL/min/1.73 m^2^, as presented in Table 3.

The studies consistently reported a high prevalence of coronary artery disease (CAD), ranging from 71.8% to 75.9%, emphasizing the significant cardiovascular risk in the diabetic population studied. Heart failure incidence varied, with reported percentages typically around 20% to 25%, except in specific heart failure subgroups where it was notably higher. Regarding the cardiovascular and mortality outcomes, Cannon et al. [21] observed a hazard ratio (HR) of 0.97 (95.6% CI, 0.85–1.11) for MACEs, indicating that ertugliflozin was noninferior to a placebo in preventing cardiovascular events. This study also reported more substantial results for heart failure-related outcomes, with HR for hospitalization due to heart failure at 0.70 (95% CI, 0.54–0.90), showcasing a significant reduction.

Dagogo et al. [24] found variable HRs for MACEs when ertugliflozin was used in combination with other diabetes medications. Notably, the HR was 0.92 (95% CI 0.790, 1.073) when used with metformin, and without metformin, the HR rose to 1.13 (95% CI 0.867, 1.480). This suggests a potential interaction effect between ertugliflozin and metformin that may enhance cardiovascular protection.

Cosentino et al. [27] and Pratley et al. [28] both reported significant reductions in heart failure hospitalizations, with Pratley et al. noting HRs of 0.72 (95% CI 0.52–0.99) for older adults (≥65 years) and 0.66 (95% CI 0.43–1.02) for those younger than 65, indicating robust protective effects across age groups. Additionally, Pratley et al. [28] highlighted ertugliflozin’s renal benefits, showing HRs that indicated reduced risks of significant declines in renal function, such as a sustained eGFR reduction of at least 40% from baseline with HRs of 0.71 (95% CI 0.47–1.09) for older adults and 0.62 (95% CI 0.43–0.91) for the younger subgroup, as presented in Table 4.

## 4. Discussion

### 4.1. Summary of Evidence

The current systematic review has elucidated several critical findings that bear significant implications for clinical practice. The most notable outcome is the substantial reduction in hospitalization for heart failure when patients are treated with ertugliflozin, as evidenced by a hazard ratio (HR) of 0.70. This finding is particularly important, considering the high morbidity and healthcare costs associated with heart failure hospitalizations in diabetic populations. The ability of ertugliflozin to reduce these hospitalizations supports its role not only as a glycemic control agent but also as a preventive strategy against one of the most severe cardiovascular complications in diabetes.

Furthermore, the additional cardiovascular benefits observed when ertugliflozin was combined with metformin, although not reaching statistical significance (HR 0.92), suggest a potential synergistic effect that could be clinically relevant. This outcome prompts consideration of the combination of these therapies as a standard approach in managing patients with T2DM who are at elevated cardiovascular risk. Given that metformin is already a first-line treatment in T2DM, the integration of ertugliflozin could be readily implemented, enhancing patient outcomes through combined metabolic and cardiovascular risk reduction.

The subgroup analysis revealing significant benefits in patients with existing heart failure (HR 0.53) further highlights ertugliflozin’s role in this high-risk subgroup. These findings suggest that ertugliflozin not only prevents the worsening of heart failure but may also contribute to its management, marking a pivotal shift in how clinicians approach the intersection of diabetes and heart failure. As heart failure and diabetes frequently coexist, introducing ertugliflozin could significantly alter clinical outcomes for these patients, potentially reducing both hospitalization rates and overall cardiovascular morbidity.

Moreover, the consistent effect across diverse international populations emphasizes the global applicability of ertugliflozin, reinforcing its potential benefit in various ethnic and racial groups. This widespread efficacy supports broad clinical adoption and suggests that ertugliflozin can be an essential component of cardiovascular risk management in diabetes care protocols globally, offering a unified strategy to tackle the dual challenges of glucose control and cardiovascular risk mitigation.

The ERASe trial from Austria [29], a phase III study, explored the use of ertugliflozin in reducing the ventricular arrhythmic burden in patients with reduced or midrange ejection fraction, irrespective of their diabetic status. This multicenter, randomized, double-blind, placebo-controlled trial aimed to enroll 402 patients to investigate whether daily administration of ertugliflozin (5 mg) could decrease the total burden of ventricular arrhythmias. However, the final results are still awaiting publication. Meanwhile, the study by Croteau et al. [30] provided mechanistic insights, demonstrating how ertugliflozin might exert its cardiovascular benefits by reversing diastolic dysfunction and impaired energetics, as evidenced by improvements in phosphocreatine (PCr) and the PCr/ATP ratio, and potentially through the reduction in elevated myocardial intracellular sodium in a mouse model of diabetic cardiomyopathy, which also has possible implications in the prevention of arrythmia. However, the ERASe study does have its limitations, such as the relatively limited 52-week follow-up period, as well as its focus on patients with implantable cardiac defibrillators and/or cardiac resynchronization therapy devices. This may suggest that the results may not be applicable to all heart failure patients, particularly those without such devices. Furthermore, the results include individuals from a single geographical region; thus, the trial may have limited power to generalize the results towards broader, more diverse populations.

One study that was not included in the final analysis of the current systematic review was the post hoc analysis of the VERTIS CV trial by Pandey et al. [31], revealing that ertugliflozin significantly reduced the risk of a first HHF with a hazard ratio of 0.70 (95% CI 0.54–0.90). Notably, the reduction in HHF was more pronounced in patients with EF ≤ 45% (HR 0.48, 95% CI 0.30–0.75) compared to those with higher EFs, demonstrating its greater efficacy in this subgroup. Additionally, the total HHF events also showed significant reduction, particularly among those with EF ≤ 45% (rate ratio 0.39, 95% CI 0.26–0.57). Also, regarding mortality, the hazard ratio was 0.92 (95% CI, 0.77–1.10), which indicates no significant reduction in death from all causes with ertugliflozin compared to a placebo. Overall, ertugliflozin has been shown to provide significant benefits in reducing hospitalization for heart failure and improving glycemic control; the VERTIS CV trial data did not demonstrate a statistically significant reduction in overall cardiovascular mortality. Zhang et al.’s meta-analysis [32] of ertugliflozin in type 2 diabetes patients highlighted its effectiveness in reducing glycated hemoglobin levels (weighted mean difference −0.77% for 5 mg and −0.82% for 15 mg), fasting plasma glucose, and body weight, with an added risk of genital mycotic infections.

The VERTIS RENAL study [33] evaluated the efficacy of ertugliflozin in T2DM patients with stage 3 CKD, showing modest A1C reductions at 26 weeks (least squares mean changes of −0.3% for both 5 mg and 15 mg doses compared to −0.4% for placebo). Despite some compliance issues with metformin use, ertugliflozin also led to significant improvements in body weight and fasting plasma glucose levels, demonstrating its utility even in patients with diminished renal function. On the other hand, the VERTIS CV trial [34] analyzed the effects on kidney composite outcomes in a broader diabetic population with established atherosclerotic cardiovascular disease, finding a notable reduction in the risk of severe renal outcomes (HR 0.66, 95% CI 0.50–0.88) for the exploratory composite renal endpoint. Furthermore, ertugliflozin was associated with a preservation of eGFR and a consistent decrease in the urinary albumin/creatinine ratio (UACR), which was particularly beneficial in patients with macroalbuminuria and high/very high-risk CKD.

The studies by Amin et al. and Cheng et al. provide a comprehensive examination of the cardiovascular and renal impacts of ertugliflozin in patients with type 2 diabetes mellitus. Amin et al. [35] focused on the blood pressure-lowering effects of ertugliflozin in T2DM patients with hypertension, demonstrating that all doses of ertugliflozin (1, 5, 25 mg) achieved significant reductions in 24 h mean systolic blood pressure by −3.0 to −4.0 mmHg over 4 weeks, which was comparable to the −3.2 mmHg reduction with hydrochlorothiazide (HCTZ; 12.5 mg). The drug also effectively decreased fasting plasma glucose and increased urinary glucose excretion without affecting plasma renin or urinary aldosterone levels. On the other hand, Cheng et al. [36] conducted a systematic review and meta-analysis assessing ertugliflozin’s long-term effects on renal function and cardiovascular outcomes. Their findings indicated a statistically significant reduction in estimated glomerular filtration rate (eGFR) by 0.60 mL/min/1.73 m^2^, suggesting a potential decline in renal function associated with the drug’s use over no more than 52 weeks. However, ertugliflozin did not increase the risk of major cardiovascular events like acute myocardial infarction or angina pectoris, highlighting its cardiovascular safety profile. Together, these studies affirm the beneficial effects of ertugliflozin on blood pressure and cardiovascular risk in T2DM patients, while also prompting caution regarding its potential renal effects over time.

The clinical utility of this systematic review is significant, especially in enhancing cardiovascular management strategies for patients with type 2 diabetes treated with ertugliflozin. By evaluating diverse international studies, the review substantiates ertugliflozin’s role in reducing major adverse cardiovascular events and heart failure-related hospitalizations. This evidence supports more informed therapeutic decisions, suggesting that ertugliflozin can be effectively integrated into treatment protocols to improve cardiovascular outcomes in this high-risk patient population, thus guiding personalized treatment approaches and improving overall care quality.

### 4.2. Limitations

However, this review is not without limitations. A significant concern is that all the studies conducted subgroup analyses or post hoc analyses on the same VERTIS-CV trial, which might introduce biases related to the selection and interpretation of data. Subgroup analyses, particularly when they are post hoc, can often find spurious associations that might not replicate in broader or different patient populations. Additionally, the review excluded studies that did not differentiate the effects of ertugliflozin from other SGLT2 inhibitors, potentially overlooking comparative insights that could refine the understanding of its unique benefits or risks. Critically, while our study design reflects broader clinical applications, it inherently limits our ability to definitively quantify the isolated impact of ertugliflozin. We acknowledge that this is a limitation and suggest that future research could benefit from conducting propensity analyses that adjust for the effects of additional medications. Our findings, therefore, should be interpreted with an understanding of these contextual nuances and the potential for confounding variables within the treatment regimes. A meta-analysis was not performed due to the significant heterogeneity among the included studies, which focused on various populations of diabetic patients and specific subgroups.

## 5. Conclusions

In conclusion, this systematic review provides robust evidence supporting the cardiovascular benefits of ertugliflozin in patients with T2DM, particularly in reducing hospitalizations for heart failure and possibly improving overall cardiovascular outcomes when combined with metformin. It is also important to take into consideration that up until this point, current findings still have yet to demonstrate a significant impact on overall cardiovascular mortality. These findings should encourage the incorporation of ertugliflozin into clinical practice for managing cardiovascular risk in T2DM patients. Clinicians are advised to consider these benefits when devising treatment plans for patients at high risk of cardiovascular events. Future research should aim to confirm these findings in larger, more diverse populations and explore the full potential of ertugliflozin within the broader spectrum of cardiovascular and metabolic diseases.

## Figures and Tables

**Table 1:** Study characteristics.

Number	First Author	Reference	Country	Study Year	Study Design	Study Quality
1	Cannon et al.	[23]	International	2020	Randomized clinical trial	High
2	Dagogo et al.	[24]	International	2022	Randomized clinical trial	High
3	Cherney et al.	[25]	International	2023	Randomized clinical trial	High
4	Segar et al.	[26]	International	2022	Randomized clinical trial	High
5	Cosentino et al.	[27]	International	2020	Randomized clinical trial	High
6	Pratley et al.	[28]	International	2023	Randomized clinical trial	High

**Table 2:** Characteristics of patients.

Number	First Author	Reference	Sample Size	Age (Years)	Comparison Group	BMI	Race
1	Cannon et al.	[23]	Ertugliflozin (n = 5499)	64.4	Placebo (n = 2747)	32.0 ± 5.5	White 87.8%
2	Dagogo et al.	[24]	Ertugliflozin + Metformin (n = 6286)	64.0	Without Metformin (n = 1960)	32.0 ± 5.4	White 87.6%
3	Cherney et al.	[25]	Ertugliflozin HF (n = 1605)	64.6	Ertugliflozin no HF (n = 3894), Placebo HF (n = 834), Placebo no HF (n = 1913)	32.5 ± 5.4	NR
4	Segar et al.	[26]	Ertugliflozin (n = 5499)	64.4	Placebo (n = 2747)	NR	NR
5	Cosentino et al.	[27]	Ertugliflozin HF, EF ≤ 45% (n = 319), Ertugliflozin HF, EF > 45% (n = 680)	HF, EF ≤ 45%: 64.2 years, HF, EF > 45% 64.7 years	Placebo HF, EF ≤ 45% (n = 159), Placebo HF, EF > 45% (n = 327)	HF, EF ≤ 45%: 32.1, HF, EF > 45% 32.9	NR
6	Pratley et al.	[28]	Ertugliflozin aged ≥ 65 years (n = 2775), Ertugliflozin < 65 years (n = 2722), Ertugliflozin ≥ 75 years (n = 593), Ertugliflozin < 75 years (n = 4900)	NR	Placebo aged ≥ 65 years (n = 1370), Placebo < 65 years (n = 1375), Placebo ≥ 75 years (n = 310), Placebo < 75 years (n = 2435)	Ertugliflozin aged ≥ 65 years (BMI = 31.2), Ertugliflozin < 65 years (BMI = 32.7), Ertugliflozin ≥ 75 years (BMI = 30.4), Ertugliflozin < 75 years (BMI = 32.2)	85.2–92.4%

NR—not reported; BMI—body mass index; HF—heart failure; EF—ejection fraction.

**Table 3:** Metabolic disease characteristics.

Number	First Author	Reference	Duration of Diabetes	HbA1c (Mean/Median)	Lipids *	eGFR
1	Cannon et al.	[23]	13.0 years	8.2%	Total cholesterol: 168.9 ± 46.9 mg/dL, LDL: 89.3 ± 38.5 mg/dL, HDL: 43.7 ± 12.0 mg/dL, Triglycerides: 181.4 ± 119.2 mg/dL	76.1 ± 20.9 mL/min/1.73 m^2^
2	Dagogo et al.	[24]	12.5 years	8.2%	Dyslipidemia: 75.8%	78.1 ± 20.1 mL/min/1.73 m^2^
3	Cherney et al.	[25]	12.5–13.4 years	8.2%	NR	73.5–76.8 mL/min/1.73 m^2^
4	Segar et al.	[26]	13.0 years	8.2%	LDL: 2.3(1.0) mmol/L, HDL: 1.1 (0.3) mmol/L, Triglycerides: 181.4 ± 119.2 mg/dL	76.1 ± 20.9 mL/min/1.73 m^2^
5	Cosentino et al.	[27]	HF, EF ≤ 45%: 13.4 years, HF, EF > 45% 13.0 years	HF, EF ≤ 45%: 8.2%, HF, EF > 45% 8.3%	NR	HF, EF ≤ 45%: 32.1% (30–60) years, HF, EF > 45% 26.6% (30–60)
6	Pratley et al.	[28]	9.4–13.8 years	8.0–8.4	NR	46.0–57.1% between 60 and 90 mL/min/1.73 m^2^

NR—not reported; *—lipids comprise cholesterol, low-density (LDL) and high-density (HDL) lipoproteins, and triglycerides; eGFR—estimated Glomerular Filtration Rate.

**Table 4:** Analysis of outcomes.

Number	First Author	Reference	CAD (%)	Heart Failure (%)	Other Comorbidities * (%)	Outcomes (Mortality, Major Cardiovascular Events, Hospitalization)
1	Cannon et al.	[23]	75.9%	23.7%	Peripheral arterial disease: 18.7%, Cerebrovascular disease: 22.9%	MACE: HR 0.97 (95.6% CI, 0.85–1.11), *p* < 0.001 for noninferiority. Death from cardiovascular causes or hospitalization for heart failure: HR 0.88 (95.8% CI, 0.75–1.03), *p* = 0.11 for superiority. Hospitalization for heart failure: HR 0.70 (95% CI, 0.54–0.90).
2	Dagogo et al.	[24]	75.8%	22.5%	Diabetic microvascular disease: 36.5%; Hypertension 91.3%	MACE with metformin: HR 0.92 (95% CI 0.790, 1.073); without metformin: HR 1.13 (95% CI 0.867, 1.480); MACE with insulin: HR 0.91 (95% CI 0.765, 1.092); without insulin: HR 1.06 (95% CI 0.867, 1.293); MACE with SUs: HR 1.11 (95% CI 0.890, 1.388); without SUs: HR 0.90 (95% CI 0.761, 1.060); MACE with DPP-4 inhibitors: HR 0.77 (95% CI 0.502, 1.173); without DPP-4 inhibitors: HR 1.00 (95% CI 0.867, 1.147). Hospitalization for heart failure (HHF) with metformin: HR 0.69 (95% CI 0.503, 0.940); without metformin: HR 0.71 (95% CI 0.449, 1.117).
3	Cherney et al.	[25]	NR	29.6%	NR	HF subgroup: HR 0.53 (95% CI 0.33–0.84); No-HF subgroup: HR 0.76 (95% CI 0.53–1.08).
4	Segar et al.	[26]	75.9%	23.7%	NR	Hemoglobin mediated 63.33% (95% CI 26.08–231.35) of the effect on the risk of hospitalization for heart failure when considering weighted average changes. Hematocrit mediated 40.0% (95% CI 10.61–151.17) of the effect on the risk of hospitalization for heart failure in early time period changes.
5	Cosentino et al.	[27]	HF, EF ≤ 45%: 96.9%, HF, EF > 45% 94.4%	HF, EF ≤ 45%: 1.9%, HF, EF > 45% 3.8%	HF, EF ≤ 45%: 67.3% NYHA III, HF, EF > 45% 64.3% NYHA III	Total HHF and CV death events HR = 0.83 (0.72–0.96); ertugliflozin vs. placebo 0.70 (0.54–0.90) ertugliflozin: 0.75, placebo: 1.05; ertugliflozin 5 mg vs. placebo 0.71 (0.52–0.97); ertugliflozin 5 mg: 0.75, placebo: 1.05; ertugliflozin 15 mg vs. placebo 0.68 (0.50–0.93) ertugliflozin 15 mg: 0.72, placebo: 1.05.
6	Pratley et al.	[28]	71.8–84.4%	20.7–27.1%	36.2–40.8%	Ertugliflozin versus placebo was associated with reductions in the risk of hospitalization for heart failure (≥65 years: HR 0.72, 95% CI 0.52–0.99; <65 years: 0.66, 0.43–1.02), the prespecified kidney composite outcome of a doubling in serum creatinine (≥65 years: 0.84, 0.60–1.17; <65 years: 0.78, 0.55–1.10), and the prespecified exploratory kidney composite outcome of sustained eGFR reduction of at least 40% from baseline (≥65 years: 0.71, 0.47–1.09; <65 years: 0.62, 0.43–0.91).

NR—not reported; CI—confidence interval; HR—hazard ratio; *—other comorbidities include cerebrovascular disease, peripheral arterial disease, coronary revascularization; stroke; CAD—coronary arterial disease; MACEs—major adverse cardiovascular events; SU—sulfonylureas; DPP—dipeptidyl peptidase.

## Data Availability

The original contributions presented in the study are included in the article; further inquiries can be directed to the corresponding author.
